# Kindness to Animals

**Published:** 1890-08

**Authors:** 


### KINDNESS TO ANIMALS.

School is dismissed ; the orderly children have passed into the hall. Suddenly shrieks are heard, and cries of, "Kill it ! Kill it !" The teacher flies to the door. She sees a miniature mob, and the victim—a defenceless mouse ! Commands and pleas are unheard ; the work is accomplished, and twenty-five joyous boys gloat over the lifeless body.

A wasp comes sailing through the school-room, a spider spins down from the wall,—"Kill it ! Kill it !" is heard from every side. Destruction marks the schoolboy's course. It is his delight to snap off young trees growing by the roadside ; bugs and butterflies must be held up by the legs and wings until these members drop off ; he has not the slightest scruple about scaling fish alive and stringing "bobs ;" and ant-hills were made for no other purpose than as marks for him to plant his feet upon. You need not be surprised to hear of his attending cock fights, dog fights, or any kind of a fight.

How can the teacher remedy this evil? Thoughtless ignorance is the cause of ninth-tenths of the cruelty. The thoughtless boy does not know the value of these little living things, and their place in creation. Woe to that creature smaller than himself. "*Ich bin gross und du bist klein,*" is the controlling principle of his action, as well as of the animals in the fable ; in plain English, "I am large, and you are small," and we might add, "therefore I shall step on you." Who has a better opportunity than the teacher to give him a new way of thinking?

There are Bands of Mercy in England and America, and the boys and girls who form the societies promise to do all they can to protect animals from cruel usage. Many of these are in connection with Public and Sunday Schools. The teachers have charge of the meetings, and make them interesting by songs, music and recitations. More than five thousand French schools give regular lessons on topics bearing on this subject. A noted French teacher, who had been instructing the children in kindness to animals, said : "This work has had the best influence on their lives and character."

The day is full of opportunities. If the heart of the teacher is right, the dead mouse and the wasp will furnish topics for eloquent and instructive talks. Men are but children grown. It is but a step from the crushing of these little creatures, endowed with all the organs of life, to that more exquisite torture of human beings, and the trampling of human rights. Respect for the lower forms of life is to be taught, before that wonderful thing we call character will come up fair and symmetrical.

Preaching will do but little good. The sentiment must be an intelligent one. The subject must be made interesting and instructive. Object lessons in zoology are received with the greatest interest ; and abundant material for such is supplied in the supplementary reading books and school papers and magazines. Where the objects themselves are not available, pictures may be used.

But the study of structure and habits must lead to something higher. Make the child realize that the life of the animal is dear to it. Show what the attractions of life are,—how the home, way of living and family ties make life pleasant. In these animal studies, instill the principle that the weaker are not to be driven to the wall, but defended. Encourage children to have pet animals. Out of two thousand criminals in American prisons it was found that only twelve had any pet animal in childhood. Keep working. It takes time to awaken delicate sensibilities, and sometimes there are none to awaken ; but one day you will feel gratified to hear indignant voices in the school yard saying: "Let it alone! What do you want to kill it for?"—*The Fountain.*

